# Immunotherapeutic Blockade of CD47 Increases Virus Neutralization Antibodies

**DOI:** 10.3390/vaccines13060602

**Published:** 2025-05-31

**Authors:** Lamin B. Cham, Thamer A. Hamdan, Hilal Bhat, Bello Sirajo, Murtaza Ali, Khaled Saeed Tabbara, Eman Farid, Mohamed-Ridha Barbouche, Tom Adomati

**Affiliations:** 1 Department of Microbiology, Immunology and Infectious Diseases, College of Medicine and Health Sciences, Arabian Gulf University, Manama 26671, Bahrain; 2 Department of Clinical Medicine, Aarhus University, 8220 Aarhus, Denmark; 3 Department of Infectious Diseases, Aarhus University Hospital, 8220 Aarhus, Denmark; 4 Department of Basic Dental Sciences, Faculty of Dentistry, Al-Ahliyya Amman University, Amman 19328, Jordan; 5 Medical Faculty Otto-von-Guericke, University of Magdeburg, 39120 Magdeburg, Germany; 6 Department of Anatomy, College of Medicine and Health Sciences, Arabian Gulf University, Manama 26671, Bahrain; 7 Internal Medicine IV, Oncology/Hematology, Martin-Luther-University Halle-Wittenberg, 06108 Halle (Saale), Germany; 8 Laboratory of Experimental Medicine and Pediatrics, University of Antwerp, B-2610 Antwerp, Belgium

**Keywords:** CD47, VSV, immunotherapy, antibodies, vaccines

## Abstract

Background/Objectives: CD47 is a cell surface glycoprotein moderately expressed in healthy cells and upregulated in cancer and viral infected cells. CD47’s interaction with signal regulatory protein alpha (SIRPα) inhibits phagocytic cells and its interaction with thrombospondin-1 inhibits T cell response. Experimental evidence has revealed that the blockade of CD47 resulted in the increased activation and function of both innate and adaptive immune cells, therefore exerting antitumoral and antiviral effects. Recent studies have shown that the combination of vaccines and immune checkpoint inhibitors could be a promising approach to increasing vaccine immunogenicity. Here, we investigated the vaccinal effect of anti-CD47 antibodies and discussed the possibilities of combining anti-CD47 treatments with vaccines. Methods: Using vesicular stomatitis virus (VSV), a widely used replication-competent vaccine vector, we evaluated the impact of the immunotherapeutic blockade of CD47 on cellular, humoral, and protective immunity. We infected C57BL/6 mice with VSV, treated them with anti-CD47 antibodies or an isotype, and evaluated the total immunoglobulin (Ig), IgG neutralizing antibodies, B cell activation, CD8+ T cell effector function, and survival of the mice. Results: We found that the treatments of anti-CD47 antibodies led to significantly increased Ig and IgG neutralizing antibody levels compared to the isotype treatment. Flow cytometric analysis of B cells revealed no difference in the number of circulating B cells; however, we observed an increased surface expression of CD80 and CD86 in B cells among anti-CD47-treated mice. Further analysis of the impact of CD47 blockade on T immunity revealed a significantly higher percentage of IFN-γ^+^ CD4 and IFN-γ^+^ CD8 T cells in anti-CD47-treated mice. Upon infecting mice with a lethal VSV dose, we observed a significantly higher survival rate among the anti-CD47-treated mice compared to control mice. Conclusions: Our results indicate that anti-CD47 treatment induces a stronger cellular and humoral immune response, leading to better protection. As such, immunotherapy by CD47 blockade in combination with vaccines could be a promising approach to improve vaccine efficacy.

## 1. Introduction

CD47, an integrin-associated molecule, is a cell surface glycoprotein that is widely and moderately expressed by all healthy cells. Physiologically, hematopoietic stem cells, migrating cells, and young red blood cells exhibit an increased CD47 expression to avoid macrophage-mediated phagocytosis. Such an increase in surface expression of CD47 in these cells is done to maintain homeostasis [1,2]. However, studies have shown that cancer- and virus-infected cells upregulate CD47 as an immune evasion mechanism [3,4]. In addition, activated and functional innate and adaptive immune cells also upregulate CD47 [5], although the reason why immune cells upregulate CD47 remains unclear. CD47 is reported to interact with the signal regulatory protein alpha (SIRPα) and is expressed on macrophages, conventional dendritic cells, B cells, NK cells, and a subset of CD8 T cells [6,7,8]. CD47–SIRPα interaction leads to phosphorylation of the immunoreceptor tyrosine-based inhibitory motifs, resulting the recruitment and activation of the Src homology 2 domain-containing phosphatases SHP-1 and SHP-2 [9,10,11]. In phagocytc cells, these SHP phosphatases cleave phosphate groups from proteins containing immunoreceptor tyrosine-based activation motifs and myosin light chains, thereby inhibiting pro-phagocytic signaling and preventing rearrangements to the cytoskeleton that are required for phagocytosis [9,10,11]. Therefore, CD47–SIRPα interaction leads to cascades of molecular signaling that result into an ‘anti-phagocytic’ signal that prevents phagocytosis [2,3,11]. In addition to its interaction with SIRPa, CD47 is also shown to interact with thrombospondin-1, which is mainly expressed on T cells [12], and such an interaction inhibits T cell activation and function [13]. Therefore, taking away or disrupting an immunological checkpoint such as CD47 can impact both innate and adaptive immune response.

Anti-CD47 monoclonal antibodies have been proposed and studied as an immunotherapeutic treatment for several tumors and viral infections. The treatment of anti-CD47 has been shown to increase the activation and function of macrophages, dendritic cells, and T cells, therefore leading to antitumoral and antiviral effects [3,14,15,16]. However, the effect of CD47 blockade in B cells and humoral immunity and how anti-CD47 antibodies could be amendable in vaccine advancement remain understudied. Considering the crucial role of an effective vaccine in the prevention of emerging and recurrent viral infections, the improvement of vaccine efficacy is of paramount importance. Several vaccine adjuvants have been developed and administered as a booster to increase vaccine efficacy. Recent years, the use of immune checkpoint inhibitors has been tested to increase vaccine efficacy and immunogenicity [17].

In this study we used vesicular stomatitis virus (VSV), a widely used replication-competent vector, to evaluate the ability of anti-CD47 treatment to induce a strong B cell and antibody response. C57BL6 mice were infected with VSV and treated with or without anti-CD47 antibodies. Total Ig and IgG neutralization levels as well as B and T cells’ effector function were evaluated. We found that anti-CD47 treatment induced stronger virus neutralization and increased the function of B and T cells compared to the control mice. These findings suggest a potential use of anti-CD47 antibodies in combination with vaccines to boost the host immune response and improve vaccine immunogenicity.

## 2. Materials and Methods

### 2.1. Mice and Ethical Approval

Inbred C57BL/6J (WT) mice were purchased from Jackson Laboratory. All experiments were performed using mice older than 8 weeks of age housed in ventilated cages and the health status of the mice was checked daily. All animal handling and experimental procedures complied with ethical standards, as approved by the relevant authorities (Landesamt für Natur, Umwelt und Verbraucherschutz Nordrhein-Westfalen, license 84-02-04.2013.A242, in accordance with the German Animal Welfare Act (Tierschutzgesetz), as well as EU Directive 2010/63/EU and FELASA guidelines. Animals were euthanized using cervical dislocation methods.

### 2.2. Virus

Vesicular stomatitis virus, Indiana strain (VSV-IND, Mudd–Summers isolate), was originally obtained from Professor D. Kolakofsky (University of Geneva, Switzerland). The virus was propagated in BHK-21 cells at a multiplicity of infection (MOI) of 0.01. Virus titers were determined and subsequently plaque-purified on Vero cells.

### 2.3. Anti-CD47 Monoclonal Antibodies

The mouse IgG 1 anti-mouse/human/rat CD47 (MIAP 410) (Catalog#: BE0283) and rat IgG2a anti-mouse CD47 (MIAP 301) (Catalog#: BE0270) were utilized. Both antibodies were purchased from Bio X Cell. Mice were intraperitoneally treated with 100 µg of anti-CD47 antibodies or an isotype. Mice were treated with anti-CD47 antibodies or an isotype at day 2, day 3, and day 4 post-VSV infection.

### 2.4. Neutralization Antibody Assay

C57BL/6 mice were infected intravenously with 2 × 10^6^ PFU VSV and treated intraperitoneally with 100 µg of anti-CD47 antibodies or an isotype (control) at 2, 3, and 4 days post-infection. Serum samples were extracted on day 2, 4, 6, 8, 10, and 12 dpi from both anti-CD47- and isotype-treated mice. Prior to evaluating the total immunoglobulin neutralization levels, serum samples were prediluted 1:40 in DMEM supplemented with 2% FCS and the complement system was inactivated by incubation at 56 °C for 30 min. To assess total immunoglobulin (Ig; including IgM and IgG) neutralization levels, sera were titrated in two-fold serial dilutions over 12 steps and incubated with 500 PFU of VSV. After 90 min of incubation at 37 °C, the virus–serum mixtures were applied to Vero cell monolayers. Following a 1 h adsorption period, an overlay medium was added and plates were further incubated for 24 h at 37 °C. Viral plaques were visualized and counted after crystal violet staining. To analyze the IgG neutralization, serum samples were preincubated with beta-mercaptoethanol (0.1 M), and this was done to remove other immunoglobulins in the serum. Similarly, serum samples were incubated with 500 PFU VSV and transferred to Vero E6 cells and overlaid with methylcellulose. Plaques were visualized via crystal violet staining. Antibody titers (both total Ig and IgG) were presented as two- or three-fold dilution steps (log_2_ and log_3_) times the predilution factor (that is, ×40).

### 2.5. Flow Cytometry

To analyze the B cell response, blood samples collected from infected C57BL/6J mice treated with or without anti-CD47 antibodies. Blood samples were lysed with RBC lysing buffer and washed. Blood cells were stained with CD19 (eBio1D3, 47-0193-82), CD11c (N418), CD11b (M/70), CD86 (GL1), and CD80 (16-10A1) at 4 °C for 30 min and then washed with FACS buffer and analyzed using flow cytometry. Stained cells were acquisitioned on BD LSRFortessa™ cell analyzer (BD Bioscience, Franklin Lakes, NJ, USA) and data were analyzed with FlowJo software (V10.1, FlowJo LLC., Ashland, OR, USA).

### 2.6. Intracellular Cytokine Staining

For intracellular cytokine staining, smashed splenocytes from VSV-infected mice were cultured in 5% FCS DMEM medium supplemented and incubated with 5 µg/mL VSV peptides (p8 and p52) for 1 h at 37 °C. After 1 h, brefeldin A (25 µg/mL; B7651; Sigma, Burlington, MA, USA) was added. Following incubation, cells were further incubated for 4 h at 37 °C. After a total of 5 h, samples were washed with FACS buffer and stained with surface markers CD8a (clone 53-6.7) or CD4 (clone GK1.5). Cells were incubated at 4 °C for 30 min, then fixed with 2% formalin in PBS at room temperature for 10 min. After an additional washing step, intracellular staining for IFN-γ (clone XMG 1.1, eBioscience, San Diego, CA, USA) was performed in FACS buffer containing 0.1% saponin (S4521; Sigma) for 30 min at 4 °C. Cells were subsequently washed and analyzed by flow cytometry. Acquisition was performed using a BD LSRFortessa™ cell analyzer (BD Biosciences) and data were analyzed with FlowJo software (FlowJo LLC., Ashland, OR, USA).

### 2.7. Statistical Analysis

Data are presented as means ± standard error of the mean (SEM). Unpaired Student’s *t*-tests were performed to assess statistically significant differences between groups. *p* values less than 0.05 were considered statistically significant. Statistical analyses and graphical representations were generated using GraphPad Prism, version 10.03 (GraphPad Software, USA).

## 3. Result

**Figure 1:**
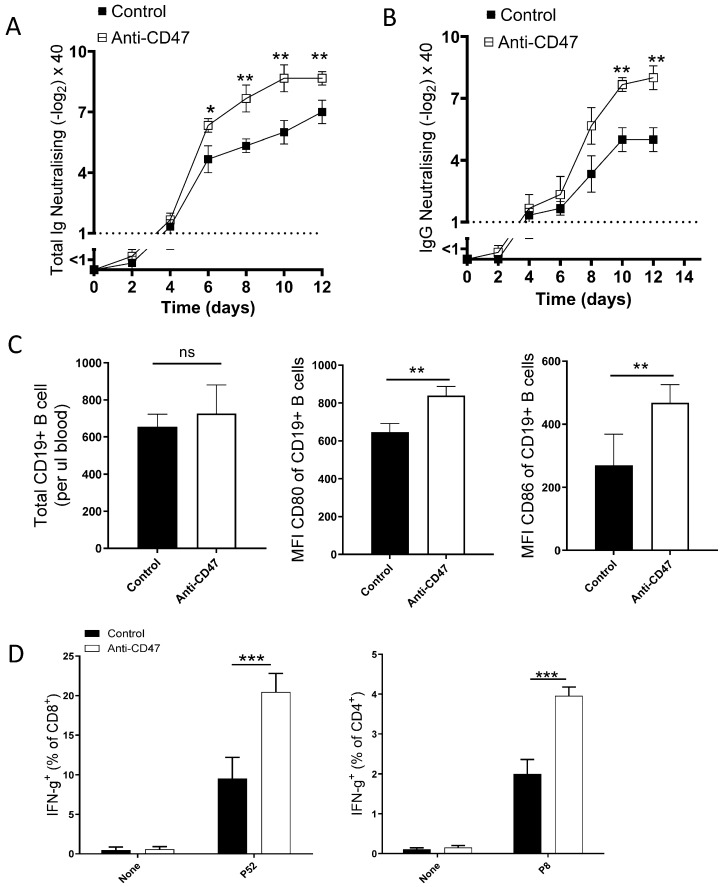
Anti-CD47 treatment enhances cellular and humoral immune response. C57BL/6 mice were intravenously infected with 2 × 10^6^ PFU VSV and treated with anti-CD47 antibodies or control (isotype) at 2, 3, and 4 dpi. (**A**) Total Ig neutralizing and (**B**) VSV-specific IgG neutralizing antibody levels at 2, 4, 6, 8, 10, and 12 dpi. (**C**) Absolute number of circulating B cells and CD80 and CD86 expression levels. (**D**) Percentage of IFN-γ^+^ CD4 and IFN-γ^+^ CD8 T cells. The data shown were confirmed in two independent experiments (n = 6) and are shown as mean ± SEM. The statistical comparison of infected vs. control mice was performed using Student’s *t*-test (* *p* < 0.05, ** *p* < 0.01, *** *p* < 0.001.

The use of immune checkpoint inhibitors (ICIs) has become a predominant therapy for cancer and certain infectious diseases [18,19]. ICIs elicit a strong host immune response without central tolerance or off-target effects [20]. Several studies have explored the possibilities of replacing the use of immunological adjuvants with ICIs during immunization. The use of ICIs in combination with antitumor vaccines been has shown to induce a stronger immune response with acceptable safety and minimal toxicity compared to the single vaccine only [17,18,21]. Using VSV, a replication-competent vaccine vector, we opted to investigate the immunological effect of anti-CD47 antibodies in inducing stronger cellular and humoral immunity. To this end, we infected C57BL/6 mice intravenously with 2 × 10^6^ PFU VSV and treated mice intraperitoneally with 100 µg of anti-CD47 antibodies or a control (isotype) at 2, 3, and 4 days post-infection (dpi). Blood samples were extracted on day 2, 4, 6, 8, 10, and 12 dpi. Total immunoglobulin (Ig) and VSV-specific IgG neutralization levels were evaluated. Compared to control mice, we found that mice treated with an anti-CD47 antibody exhibited significantly higher levels of total Ig neutralizing antibody at 6 to 12 dpi (Figure 1A). We further observed a significantly increased virus-specific IgG neutralization among anti-CD47-treated mice compared to controls (Figure 1B). Next, we opted to understand the mechanisms behind the induction of increased Ig and IgG in anti-CD47-treated mice. Again, we infected C57BL/6 mice with 2 × 10^6^ PFU VSV and treated with anti-CD47 antibodies or a control (as described above) and analyzed the cellular response from the blood and spleen at 10 dpi. While there was no difference in the number of circulating B cells in the blood, we found a significantly higher expression of antigen presentation markers (CD80 and CD86) on B cells among anti-CD47-treated mice compared to control (Figure 1C). Considering the role of CD4 and CD8 T cells in vaccine immunogenicity and efficacy [22], we performed an intracellular cytokine assay to evaluate the percentage of IFN-γ-producing CD4 and CD8 T cells using VSV peptide p52 and p8, respectively. Using flow cytometry, we analyzed the percentage of IFN-γ^+^ T cells as shown in a gating strategy (Supplementary Figure S1A). Compared to the control mice, we observed a significantly higher percentage of IFN-γ^+^ CD4 and IFN-γ^+^ CD8 T cells in anti-CD47-treated mice (Figure 1D). Overall, our findings indicate that the treatment of anti-CD47 antibodies as an ICI can induce a stronger B cell activation and function, stronger virus neutralization levels, and stronger T cell effector function.

**Figure 2:**
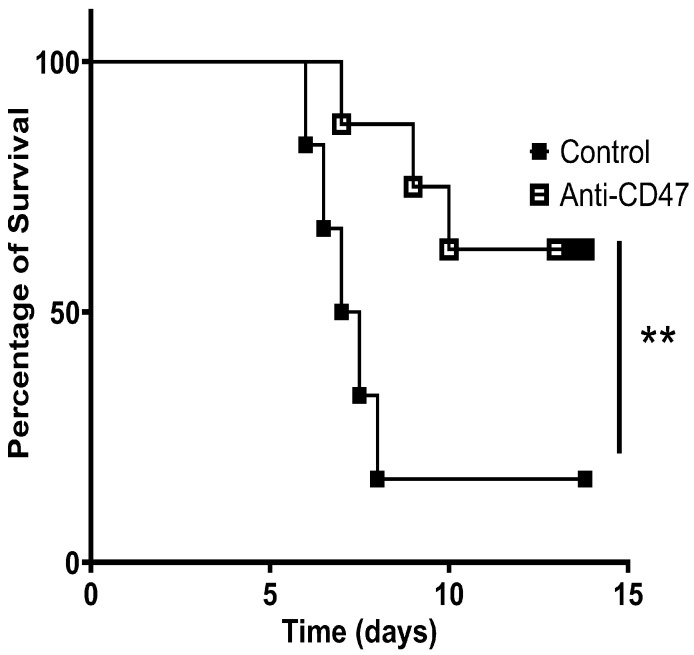
Anti-CD47 treatment improved survival rate after infection with lethal VSV dose. C57BL/6 mice were intravenously infected with 2 × 10^8^ PFU VSV and treated with anti-CD47 antibodies or a control (isotype) at 2, 3, and 4 dpi. Survival rate among anti-CD47-treated mice compared to control mice. The data shown were confirmed in two independent experiments (n = 8). The statistical comparison of infected vs. control mice was performed using Student’s *t*-test (** *p* < 0.01).

Based on the observed immunological effects of anti-CD47 treatment, we further investigated whether such effects have a lasting benefit. Here, we infected C57BL/6 mice with a lethal VSV dose (2 × 10^8^ PFU) and treated mice intraperitoneally with 100 μg of anti-CD47 antibodies or a control (isotype) at 2, 3, and 4 dpi and monitored their survival. We found minimal weight loss (Supplementary Figure S1B) and a significantly higher survival rate among mice treated with anti-CD47 antibodies compared to the control mice (Figure 2). Together, our data revealed that the treatment of anti-CD47 antibodies elicited a robust cellular and humoral immunity, leading to the protection of mice against VSV pathology and death.

## 4. Discussion

Historically, effective vaccination has saved millions of lives and made enormous contributions in solving global public health threats. With the emergence and reemergence of infectious pathogens, the development of novel approaches to increasing vaccine efficacy and effectiveness is crucial. One of the approaches that has yielded promising potential is the combination of immunotherapeutic agents with vaccines. Our study attempted to explore the potentials of using anti-CD47 antibodies in combination with a vaccine-like virus. Here, we infected mice with a replication-competent vaccine vector, then treated them with anti-CD47 antibodies or an isotype and evaluated the vaccinal effects. We found that the treatment of anti-CD47 antibodies significantly increased Ig and IgG neutralization levels compared to VSV only. Additionally, the anti-CD47-treated mice exhibited increased activation and effector function of T and B cells. The immunological effect of the anti-CD47 treatment was observed to protect mice from disease progression and death.

Mechanistically, the cellular and molecular mechanisms of CD47 interaction and its role in B cell activation and function remain poorly understood. However, several studies have shown that CD47 is highly expressed in B cells and plays an immuno-inhibitory role in inducing B cell response [23,24,25]. CD47 is shown to negatively regulate germinal B cells and plasma cells [25]. Like macrophages and dendritic cells, B cells can phagocytose and participate in antigen presentation. Antigenic-loaded B cells interact with CD4^+^ and CD8^+^ T cells, thus leading to their activation and function [26]. Considering the well-known inhibitory effect of CD47 on phagocytosis and antigen-presenting cells [9,10,11], we showed that a lack of CD47 increases B cells’ antigen presentation potential. These findings are consistent with other studies suggesting that CD47-deficient B cells are better in antigen presentation compared to CD4 T cells and therefore help in B cells’ differentiation and antibody production [23,26].

For the past decades, multiple ICIs and vaccines have been developed, approved, and used in both pre-clinical and clinical studies. However, combinations of ICIs and vaccines have been recently reported to prime, expand, and facilitate effective immunization [17,18,21,27,28]. Several cancer studies have reported a stronger immunological response among vaccinated cancer patients on immunotherapy compared to patients that received a vaccine only [27,28,29,30]. For instance, immunotherapy using the anti-PDL1 antibody in combination with a vaccine revealed evidence of safety, induction of a stronger immunological response, and the suppression of gastrointestinal tumors compared to those that received the anti-PDL1 treatment or the vaccine only [27,29]. In the infectious disease realm, experimental studies have provided evidence of safety in combining inactivated influenza and mRNA SARS-CoV-2 vaccines with ICI therapy [21,31,32]. These studies highlighted that the combination of an ICI with viral vaccines does not exacerbate immune-mediated adverse effects. Additionally, a stronger immunological response was observed among those that received the ICI with the vaccine compared to the vaccine only [31,32,33]. Notably, the efficacy of vaccines administered alongside ICIs in preventing other infectious diseases remains poorly studied.

## 5. Conclusions

Our study complements these recent attempts to test the possibilities of combining vaccines with ICIs such as anti-CD47 antibodies. Taken together, our results demonstrate that CD47 plays an immunosuppressive role in B cells and that the immunotherapeutic blockade of CD47 led to a significantly increased B cell activation and function as well as higher levels of neutralization antibodies. The importance of our study is that anti-CD47 antibodies can be potentially used as an adjuvant to stimulate and enhance vaccine effectiveness. However, further studies are required to elucidate the safety, immunogenicity, and efficacy of combining anti-CD47 antibodies and vaccines.

## Data Availability

The original contributions presented in this study are included in the article/Supplementary Material. Further inquiries can be directed to the corresponding author(s).

## Supplementary Materials

The following supporting information can be downloaded at: https://www.mdpi.com/article/10.3390/vaccines13060602/s1, Figure S1: (A) Gating strategy for IFN-γ^+^ CD8 T cells. (B) Body weight curve of C57BL/6 mice intravenously with 2 × 10^8^ PFU VSV and mice treated with anti-CD47 antibodies or a control.
