# Supplementary figures and images for: Immunotherapeutic Blockade of CD47 Increases Virus Neutralization Antibodies
Source: Vaccines (Basel). 2025 May 31;13(6):602. doi: 10.3390/vaccines13060602 (PMC12197644; doi:10.3390/vaccines13060602)

# Supplementary Figure S1

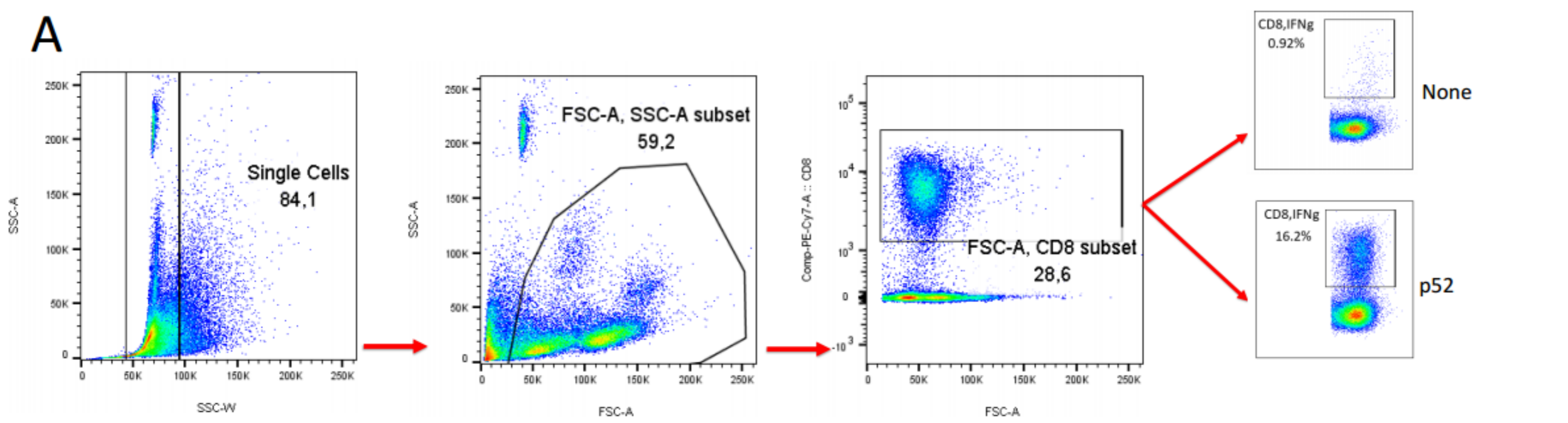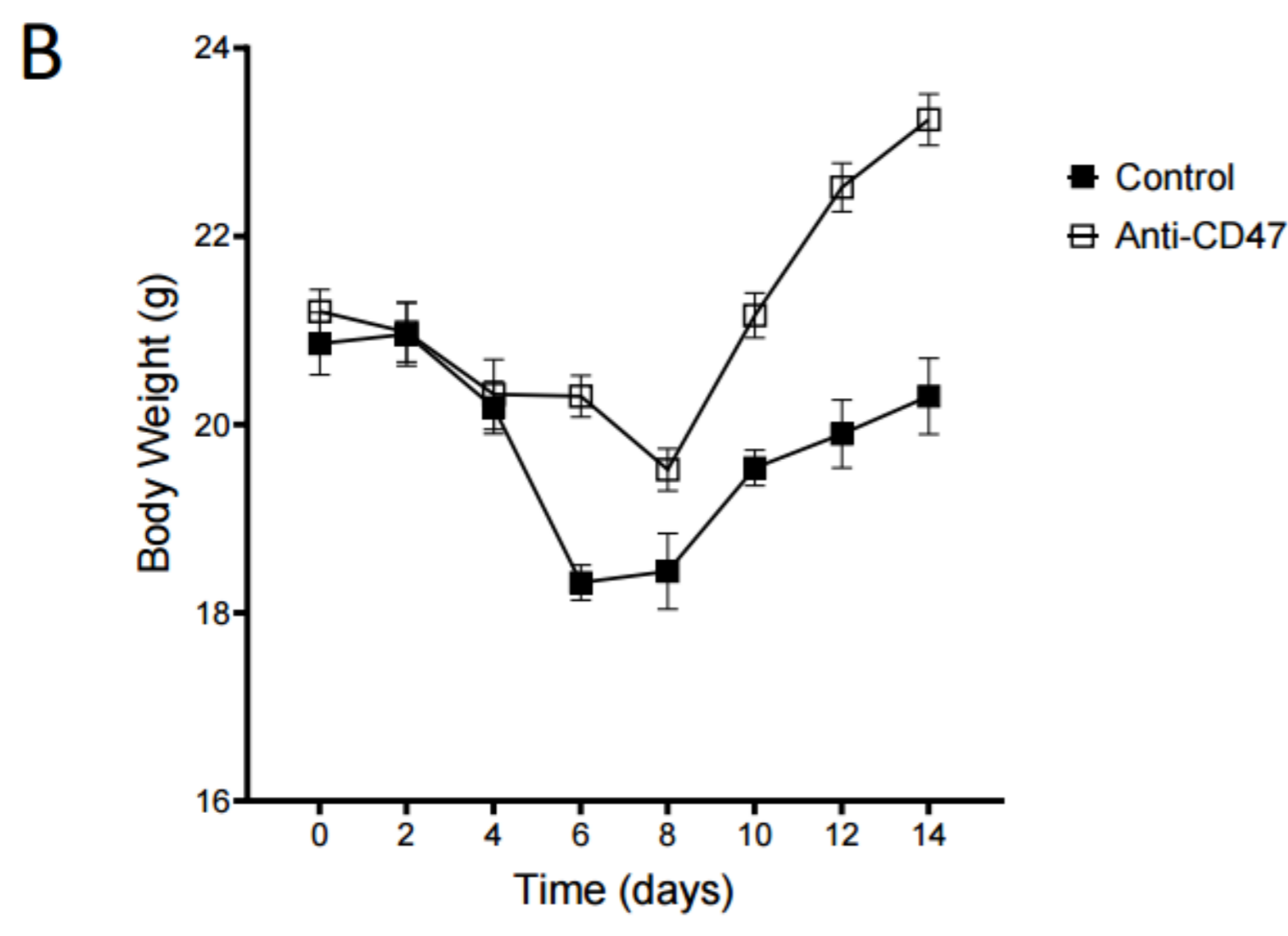

Supplement: Supplementary file 1 [file vaccines-13-00602-s001.zip › vaccines-3574631-supplementary.pdf]
